# Prompt neuroendoscopic intervention prevented hydrocephalus after ventricular rupture of the brain abscess. A case report

**DOI:** 10.1186/2045-8118-12-S1-P47

**Published:** 2015-09-18

**Authors:** Toshimasa Shin, Hisayuki Murai, Hirokazu Sunaoka, Naokatu Saeki

**Affiliations:** 1 Chiba University Graduate School of Medince, Japan

## Introduction

Ventricular abscess is one of the worst complications of the brain access. Despite standard treatments such as continuous ventricular drainage and antibiotic agents, ventricular abscess frequently causes hydrocephalus or isolated ventricles. And its morbidity and mortality is high. In the present article, a case of brain abscess with intraventricular rupture successfully treated with prompt neuroendoscopic ventricular lavage is reported.

## Case presentation

An eleven years old male was admitted to our hospital with headache and fever. Magnetic resonance imaging (MRI) showed right frontal and left parietal mass lesion. The right frontal mass lesion was 22 mm, and the left was 24mm in diameter. Diffusion weighted MRI showed high intensity areas in both lesion and frontal lesion was closely located to the lateral ventricle. Gadolinium contrast enhanced MRI showed ring enhancement of both lesions. Despite 4 days use of antibiotics, the volume of lesions expanded and ventricular abscess became evident on MRI. He became restless and confused, so we performed drainage of both abscess and ventricular lavage with flexible neurovideoscope (Olympus VEF-V). At a day after the operation, he became alert and calm. The lesions disappeared on MRI after 2 months and he was discharged. His chest computed tomography showed two pulmonary nodules of arteriovenous malformation (AVM) which was successfully emobolized later.

## Conclusions

We presented a case of successfully treated ventricular abscess with prompt ventricular lavage. It avoided hydrocephalus or ventricular shunt surgery. However this report is preliminary and we need more cases to confirm its efficacy and safety, prompt neuroendoscopic intervention to ventriculitis is seemed to be promising.

